# Remodeling of Monoplanar Purkinje Cell Dendrites during Cerebellar Circuit Formation

**DOI:** 10.1371/journal.pone.0020108

**Published:** 2011-05-31

**Authors:** Megumi Kaneko, Kazuhiko Yamaguchi, Mototsugu Eiraku, Motohiko Sato, Norio Takata, Yoshimoto Kiyohara, Masayoshi Mishina, Hajime Hirase, Tsutomu Hashikawa, Mineko Kengaku

**Affiliations:** 1 Laboratory for Neural Cell Polarity, RIKEN Brain Science Institute, Wako, Saitama, Japan; 2 Laboratory for Neural Architecture, RIKEN Brain Science Institute, Wako, Saitama, Japan; 3 Laboratory for Memory and Learning, RIKEN Brain Science Institute, Wako, Saitama, Japan; 4 Hirase Research Unit, RIKEN Brain Science Institute, Wako, Saitama, Japan; 5 Institute for Integrated Cell-Material Sciences, Kyoto University, Sakyo-ku, Kyoto, Japan; 6 Department of Molecular Neurobiology and Pharmacology, Graduate School of Medicine, University of Tokyo, Bunkyo-ku, Tokyo, Japan; Institut de la Vision, France

## Abstract

Dendrite arborization patterns are critical determinants of neuronal connectivity and integration. Planar and highly branched dendrites of the cerebellar Purkinje cell receive specific topographical projections from two major afferent pathways; a single climbing fiber axon from the inferior olive that extend along Purkinje dendrites, and parallel fiber axons of granule cells that contact vertically to the plane of dendrites. It has been believed that murine Purkinje cell dendrites extend in a single parasagittal plane in the molecular layer after the cell polarity is determined during the early postnatal development. By three-dimensional confocal analysis of growing Purkinje cells, we observed that mouse Purkinje cells underwent dynamic dendritic remodeling during circuit maturation in the third postnatal week. After dendrites were polarized and flattened in the early second postnatal week, dendritic arbors gradually expanded in multiple sagittal planes in the molecular layer by intensive growth and branching by the third postnatal week. Dendrites then became confined to a single plane in the fourth postnatal week. Multiplanar Purkinje cells in the third week were often associated by ectopic climbing fibers innervating nearby Purkinje cells in distinct sagittal planes. The mature monoplanar arborization was disrupted in mutant mice with abnormal Purkinje cell connectivity and motor discoordination. The dendrite remodeling was also impaired by pharmacological disruption of normal afferent activity during the second or third postnatal week. Our results suggest that the monoplanar arborization of Purkinje cells is coupled with functional development of the cerebellar circuitry.

## Introduction

Dendrites show remarkable diversity in morphology depending on neuronal function in the brain. The size and pattern of dendritic arbors affect the number and types of synaptic inputs. Moreover, the complexity of dendritic structures greatly influences the information processing of neurons [1], [2], [3]. Defects in dendritic patterning are often accompanied with mental retardation and neurological disorders [4].

The establishment of the dendritic tree is a highly dynamic process that involves addition, extension, stabilization and pruning of branches. Recent progress indicates that dendritic growth is regulated by both activity-dependent and activity-independent mechanisms. Activity-independent mechanisms include cell-intrinsic programs and environmental cues, which have profound effects on the determination of the basic pattern of dendrites in early brain development [5], [6]. In contrast, activity-dependent mechanisms are more critically important for dendritic growth in circuit reconstruction during later brain development. For instance, retinal ganglion cells undergo dynamic dendritic and synaptic remodeling during postnatal development, which is disturbed by inhibition of visual inputs or synaptic activity of afferent interneurons [7], [8]. Although activity-dependent dendritic remodeling has been implicated in neurons in sensory systems where afferent inputs can be easily manipulated, relatively little is known about the precise relationship between afferent activity and dendritic remodeling in other systems in the brain [5], [9].

The cerebellar Purkinje cell is a unique neuron that has a very large and highly branched dendritic tree with planar expansion in all three spatial dimensions. The fan-shaped dendrites align along the parasagittal axis coding functional subdivisions of cerebellar neural circuits [10], [11]. Mature Purkinje cell dendrites receive two major excitatory inputs; a single climbing fiber axon (CF) from the inferior olive extend along the flat Purkinje dendrites in a sagittal plane [12]; 10^5^–10^6^ parallel fiber axons (PFs) of cerebellar granule cells traverse along the longitudinal (mediolateral) axis of the cerebellum and contact vertically to the plane of Purkinje dendrites [13], [14]. During the first postnatal week of murine development, Purkinje cells extend multiple dendrites from the cell body in random orientations. A single primary dendrite is determined during the second postnatal week, which rapidly extends and branches in a single parasagittal (translobular) plane [15]. In contrast to murine Purkinje cell dendrites that are thought to become monoplanar from the second postnatal week, earlier anatomical work using Golgi impregnation has shown that Purkinje dendrites in cat cerebellum transiently extend in two parallel sagittal planes in the second postnatal week and later confine to a single plane [16]. This morphological variation has been attributed to species difference, and the biological significance of transient biplanar arrangement of cat Purkinje cells is unknown.

Intensive studies have indicated that the dendritic growth of Purkinje cells is regulated by many extrinsic signals including steroid and thyroid hormones, neurotrophins and growth factors [17]. Involvement of afferent inputs from PFs has also been shown, since the dendritic growth and survival of Purkinje cells are severely affected in mutants and conditions that are defective in PF inputs [18], [19], [20], [21]. Earlier studies have also suggested that vertical contacts with afferent PFs are required for the flat arborization of Purkinje cell dendrites. In mice deficient in contactin, however, Purkinje cells extend seemingly normal dendrites along the sagittal axis despite a large portion of PFs being misoriented in different directions in the molecular layer, questioning the instructive role of geometric arrangement of PF inputs in patterning Purkinje cell dendrites [22]. Thus, it remains unknown what regulates the patterning of monoplanar Purkinje cell dendrites.

To understand the mechanisms of the monoplanar arborization of Purkinje cell dendrites, we analyzed the cellular morphogenesis of mouse Purkinje cells using virus-mediated gene transfer followed by three-dimensional reconstruction of confocal images of labeled cells. We demonstrate that Purkinje cells obtain a monoplanar configuration by dynamic remodeling from irregular arrangement extended in multiple sagittal planes during the third postnatal week in mice. This dendritic remodeling is parallel to the refinement of climbing fiber inputs to Purkinje cells. We propose that normal synaptic activity during postnatal development is prerequisite for the formation of monoplanar dendrites.

## Materials and Methods

### Mice

All procedures involving animals were approved by the RIKEN Experimental Animal Committee on the care and use of animals in experiments (Approval ID: H19-2B002). Mice were kept under standard conditions of feeding and lightening (12 h light/dark cycle, 22°C). Pregnant female Slc:ICR mice were purchased from Japan SLC and 50 pups of either sex were used for analysis of normal development from postnatal day 7 (P7) to P50. GluRδ2-knockout mice (University of Tokyo colony)[23] and GLAST-knockout mice [24](RIKEN Brain Science Institute colony) were of 99.99% C57BL/6 genetic background. Homozygous mutant and wildtype littermates were generated by heterozygous cross and their genotypes were determined a posteriori by polymerase chain reaction (PCR). Fourteen and 18 pups were used for GluRδ2-mutant and GLAST-mutant mice, respectively. Quantitative analyses were made using cells from 3 to 5 nonsibling pups.

### AAV construction and injection

For efficient expression in neurons, an AAV vector with a chicken beta-actin (CAG) promoter (pCAG-MCS) was made by converting the CMV promoter of pCMV-MCS (Agilent Technologies, Santa Clara, CA) to the CAG promoter excised from pCAGGS [25]. EGFP cDNA was cloned into the pCAG-MCS and AAVs were prepared by the AAV purification kit (Virapur, San Diego, CA) at 10^12^ pfu/ml. For cerebellar injections, P2 mouse pups were anesthetized by hypothermia and positioned on a stage designed for stereotaxic injections in neonatal mice. The occipital skin and muscle was cut open, and a small incision was made in the bone over the cerebellar vermis with a 27-gauge needle. The tip of a microsyringe with a 33-gauge needle (Ito, Fuji, Japan) was inserted 0.5 mm through the incision into the molecular layer of the cerebellar vermis (lobules IV–VI). One-two µl of the concentrated AAV suspension was stereotaxically injected over 60 sec. After the wound was sutured, the pups were revived at 37°C and returned to the litter.

### BDA labeling

Rhodamine conjugated BDA (Invitrogen, Carlsbad, CA) was dissolved in distilled water. For anterograde labeling of climbing fibers, 0.05 µl of 5% BDA solution was stereotaxically injected into the caudo-medial part of the inferior olive of the AAV-injected mice at P14 under Nembutal anesthesia (50 mg/kg). A glass pipette (tip diameter, ca 60 µm) connected with a 1.0 µl Hamilton syringe was introduced into the brain through the foramen magnum at an angle of 30–40 degrees caudal from the vertical [26]. In some cases, BDA was injected iontophoretically by a 7 µA positive current for 10–30 min with a protocol of 7 sec on and 7 sec off. Retrograde labeling of Purkinje cells was performed as previously described [27]. BDA was injected into the medial cerebellar nucleus at P15 and P22, and labeled Purkinje cells were assayed at P18 and P25, respectively. After the wound was sutured, the pups were revived at 37°C and returned to the litter.

### Immunohistochemistry

Mice were anesthetized with isoflurane and transcardially perfused with 4% paraformaldehyde (PFA) in phosphate buffered saline (PBS, pH 7.4). The cerebellum was then dissected and postfixed in the same fixative for 24 h at 4°C. Sagittal sections (100 µm thick) were made using a vibratome (DTK-3000, Dosaka EM, Kyoto, Japan). For VGluT2-immunostaining of CF terminals, slices were permeabilized with 0.1% Triton in PBS (PBST) and blocked with 2% skim milk (BD Biosciences, San Jose, CA) diluted in PBST. The slices were then incubated with the guinea pig anti-VGluT2 antiserum (1∶500, Chemicon) in blocking solution at 4°C overnight. After washing twice with 2% skim milk in PBS, slices were incubated with Alexa647-conjugated anti-guinea pig IgG (1∶200, Invitrogen) at 4°C overnight and mounted with an antifade kit (Invitrogen). Purkinje cells and CF terminals were visualized by natural fluorescence of virus-derived GFP and rhodamine-BDA, respectively.

### Confocal microscopy and image analyses

Single- or multi-channel image acquisition was carried out with laser-confocal microscopy LSM 5 PASCAL (Zeiss, Jena, Germany) with a 40× oil-immersion objective (numerical aperture 1.3, Zeiss) or FV1000 (Olympus, Tokyo, Japan) with a 40× dry objective (numerical aperture 0.90, Olympus) by using 488 nm (for GFP), 543 nm (for rhodamine) and 633 nm (for Alexa647) lasers. Confocal serial sections with frame size of 512×512 pixels were captured at 1-µm intervals to acquire whole cell images with 0.56 µm×0.56 µm×1.0 µm pixel dimensions. The z-series was reconstituted into a 3D image using Imaris software (version 5.0.3; Bitplane, Saint Paul, MN) in the Surpass view. The Filament tracer software (AutoDepth) was then used to generate a 3D rendering of each dendritic branch. The minimum endpoint diameter (smallest dendritic tip) was set at 1.125 µm. Renderings were individually edited for correct positioning in the third dimension relative to the actual confocal image.

## Results

### Purkinje dendrites are remodeled from multiplanar to monoplanar arrangement in the third postnatal week

The morphology of the Purkinje cell was assayed by taking advantage of an adeno-associated virus (AAV) that is known to predominantly infect Purkinje cells in the cerebellum [28]. A low titer of the GFP-carrying AAV vector was injected into the cerebellar vermis of P2 mouse pups and GFP expression was analyzed in sagittal sections at P7–P50. A large majority of GFP-expressing cells were Purkinje cells, which were sparsely distributed to all cerebellar lobules (Figure S1A, B). Adjacent Purkinje cells were occasionally labeled, whose dendrites were aligned in close but distinct sagittal planes in the adult cerebellum (Figure S1C). The three-dimensional images of labeled Purkinje cells were obtained by laser scanning confocal microscopy, and processed with IMARIS software. Purkinje cells in the straight bank region (between the sulcal fundus and gyral surface) were analyzed to avoid confusion with morphological differences in distinct subdivisions [29].

**Figure 1:**
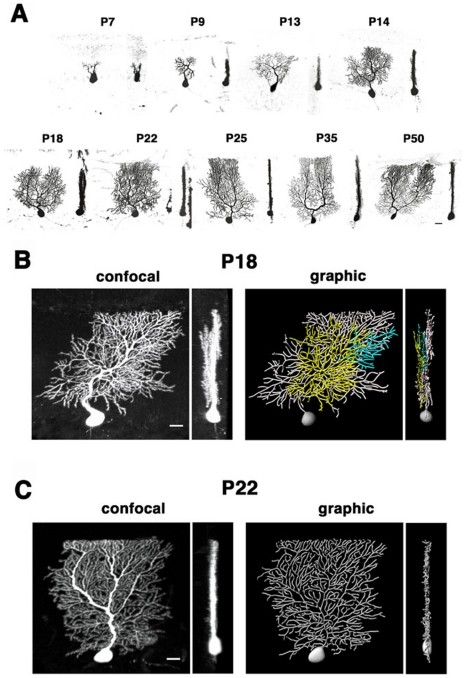
Confocal analysis of dendrite arborization in developing Purkinje cells. **A**: Sagittal (left) and coronal (right) views of developing Purkinje cells labeled with adeno-associated virus (AAV)-derived GFP. Three-dimensional images were compiled from 20–50 z-serial sections taken at 1 µm intervals. Dendritic processes at P7 orient randomly in the molecular layer. At P9 and thereafter, Purkinje cells bear a single to a few primary stem dendrites which extend branches along the sagittal axis of the molecular layer. **B**: Confocal and graphic images of typical Purkinje cells at P18. Sagittal (left panels) and coronal (right panels) views are shown. Some dendrites extrude from the sagittal plane filled by main arbors, and further branch in distinct parallel sagittal planes (pseudocolored in yellow and blue in graphic images; see also Video S1). **C**: The P22 Purkinje cell arborizes dendrites in a single sagittal plane (see also Video S2). Scale bars: 20 µm.

It has been shown that Purkinje cell somata extend multiple dendrites in random orientations during the first postnatal week of murine development. A single primary dendrite is determined during the second postnatal week, which rapidly extends and branches in a single parasagittal (translobular) plane [15], [30], [31], [32]. Consistent with previous observations, Purkinje dendrites underwent a dynamic remodeling around the first to second postnatal weeks (Figure 1A, S2). At P7, many Purkinje cells exhibited a multipolar morphology with multiple small processes emanating from the cell body (Figure S2 and Video S1). These perisomatic processes had few branches and randomly oriented in all three dimensions of the molecular layer. In the second postnatal week (P9), most Purkinje cells became flat with a single primary stem dendrite that aligned along the sagittal axis (Figure S2 and Video S2). This remodeling has been observed around P12 in rats [31], consistent with previous findings that the cerebellar cortex matures earlier in the mouse than in the rat [33]. From the third postnatal week, the primary dendrite rapidly extended and branched along the sagittal (translobular) axis of the cerebellar cortex until the beginning of the sixth postnatal week (from P14 to P35). There was little apparent change in the size and complexity of dendrites between P35 and P50.

In contrast to the general understanding that most of murine Purkinje cells extend dendrites in a single sagittal plane in the molecular layer after P10 [15], [17], we found that in the majority of the cells at P18, dendrites extended in multiple parasagittal planes (34 out of 58 cells in the lobules III-X; Figure 1B and Video S1). In these Purkinje cells, one or more secondary or higher-ordered branches extruded from the main sagittal plane of the stem dendrites, and these minor arbors further branched in distinct sagittal planes parallel to the main arbors (yellow and blue branches in Figure 1B and Video S3). The distance between the planes formed by main and minor arbors were rather constant with a mean value of 12.2±0.94 µm (mean±s.e.m., n = 12 cells). The tendency of heterotopic dendrites had already appeared at P14, but multilayered arbors were less evident due to relatively rudimentary branching at this stage (Figure S2). In contrast, the majority of the Purkinje cells at P22 and thereafter are monoplanar with dendrites in a single parasagittal plane (68 out of 104 cells in the lobules III-X at P22; Figure 1C and Video S4). The percentage of multiplanar cells showed a significant difference between P18 and P22 (p<0.05; χ^2^ test).

**Figure 2:**
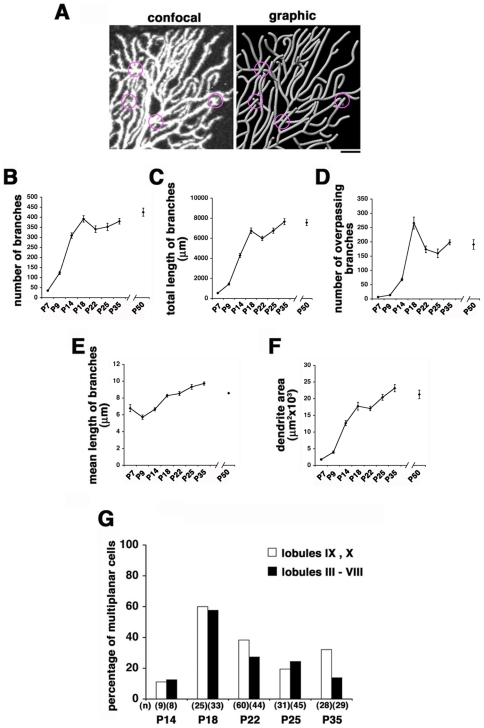
Remodeling of Purkinje dendrites in the third postnatal week. **A**: Confocal and graphic images of dendrites in a P35 Purkinje cell. Some branches extrude from the main sagittal plane and overpass other branches (magenta circles). Scale bar: 10 µm. **B–F**: Quantitative analyses of dendrite development in Purkinje cells in lobules IX and X. The number (**B**) and total length (**C**) of branches per cell rapidly increase over the first 18 postnatal days. Both the number and length significantly decrease between P18 and P22, and further increase into adulthood. **D**: Overpassing branches per cell peak at P18, sharply decrease by P22 and plateau thereafter. **E**: The mean length of each dendritic branch decreases between P7 and P9, constantly increases until P35 and then slightly decreased by P50. **F**: The total sagittal-sectional area of the molecular layer covered by the dendrite increases until P35 with a plateau between P18 and P22. Purkinje cells per data point in **C**–**G**: P7, P14–P50, n = 14; P9, n = 20. Error bars indicate s.e.m. **G**: Histogram showing the developmental change in percentages of multiplanar Purkinje cells. The percentage of multiplanar Purkinje cells peaks at P18 in both early and late maturing lobules (lobules IX, X and lobules III–VIII, respectively). The number of cells analyzed is indicated in parentheses.

A higher magnified view revealed that most branches of mature Purkinje dendrites at P35 extended in a single sagittal plane with minimal contacts or overlap (Figure 2A). In contrast, branches extending in differential sagittal planes passed over other branches without physical contact. These overpassing dendrites appeared to overlap with other branches when the three-dimensional image stacks were viewed sagittally (magenta circles in Figure 2A).

For detailed quantification of dendrite development, the number and length of all branches in developing Purkinje cells were measured. The number of overpassing dendrites in distinct sagittal planes was also measured as an index of multiplanarity. Previous anatomical work has described significant differences in the developmental timing of Purkinje cells in distinct lobules [34], [35]. Thus, Purkinje cells in the bank region in early maturing lobules (lobules IX and X) were used for quantitative assessments. We found an apparent phase shift in morphometric features around P18 in addition to a remodeling event around P9. The number and total length of all dendritic branches in a cell rapidly increased between P9 and P18, and then decreased by P22. In a subsequent phase between P22 and P35, both the number and total length of dendrites gradually increased (Figure 2B,C). Notably, the number of overpassing branches sharply peaked at P18, and then rapidly decreased by P22 and plateaued thereafter (Figure 2D). In contrast, the mean length of each dendritic branch increased constantly between P9 and P35 (Figure 2E). The total sagittal-sectional area of the molecular layer occupied by the dendrite rapidly increased by P18, plateaued between P18 and P22, and increased again after P22 (Figure 2F).

We also counted multiplanar Purkinje cells during development. The Purkinje cell with second or higher ordered branches that further branched in distinct sagittal plane(s) and at least partly overlapped with the main arbor in a sagittal view (those making overpassing branches) was defined as multiplanar. The percentage of multiplanar cells sharply peaked at P18 and decreased by P25 in parallel with the number of branch overpasses (Figure 2D,G). Indeed, close observation revealed that multiplanar arborization of the dendrites caused a significant increase in branch overpasses at P18. The dramatic change in dendritic configurations over the interval between P18 and P25 was also observed in the later maturing lobules (lobules III–VIII), suggesting that the dendritic remodeling is a common phenomenon in all cerebellar folia (Figure 2G).

The measurements of cellular morphology in above data were made using Purkinje cells labeled with AAV-derived GFP. This raises concern that the multiplanar structure might be an abnormal phenotype caused by viral infection or phototoxicity to the observed cells. To address this issue, we retrogradely labeled Purkinje cells by injecting a neuroanatomical tracer biotinylated dextran amine (BDA) into the medial cerebellar nucleus. The percentage of multiplanar Purkinje cells revealed by tracer labeling was comparable to AAV-infected Purkinje cells (Figure S3; 52%, n = 50 cells, P18 vs. 26%, n = 38 cells, P25; p = 0.062; χ^2^ test). These results strongly suggest that murine Purkinje dendrites undergo dynamic remodeling from multiplanar to monoplanar configurations around P18-P25.

### Multiplanar Purkinje dendrites are associated by climbing fibers of adjacent Purkinje cells

We hypothesized that the spatiotemporally restricted rearrangement of Purkinje dendrites might be correlated with cerebellar circuit formation during late postnatal development. Among excitatory inputs to Purkinje cells, we focused on climbing fiber (CF) terminals from the inferior olive which run along the flat Purkinje dendrites in a sagittal plane [12].

**Figure 3:**
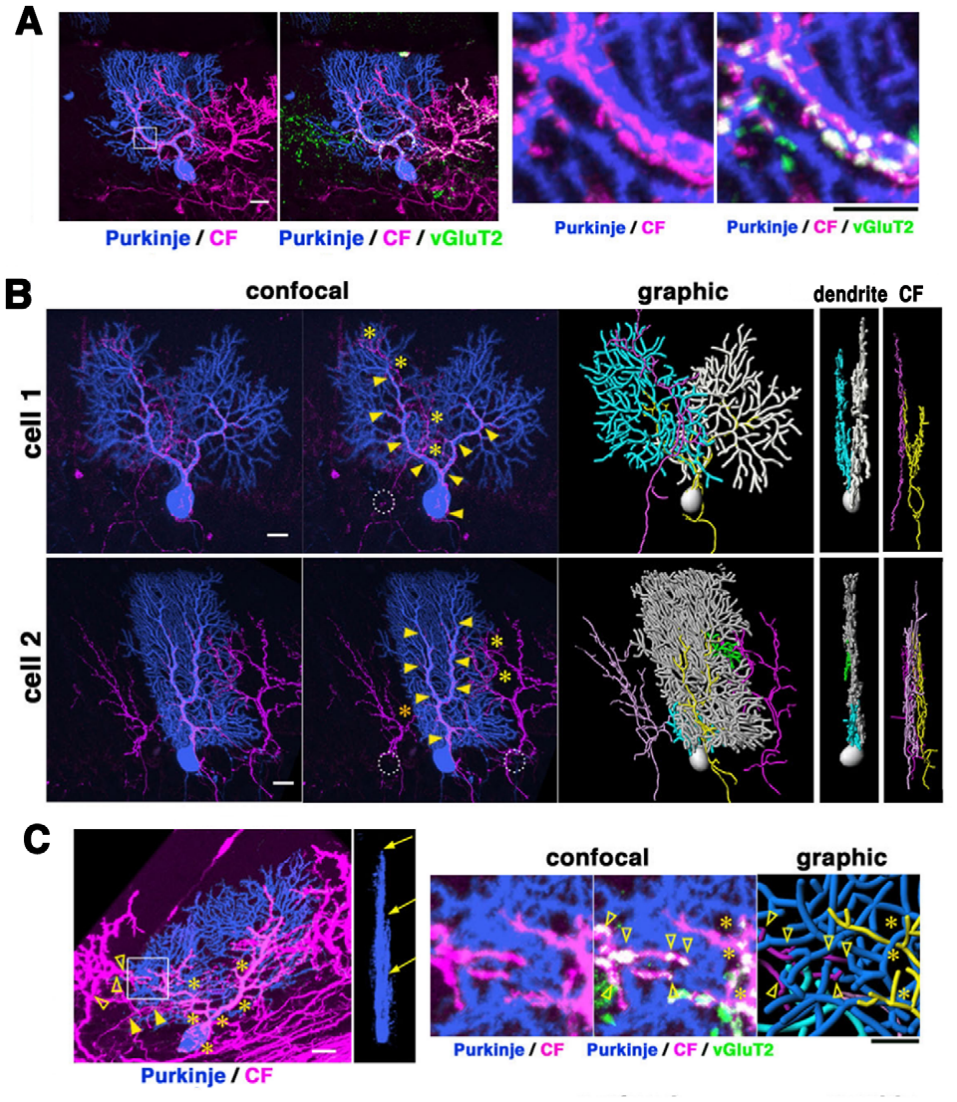
Multiple CF associations to multiplanar dendrites of Purkinje cells. **A**: Triple fluorescence for AAV-GFP-infected Purkinje cells (pseudocolored in blue), BDA-labeled CFs (magenta) and vesicular glutamate transporter VGluT2 (green). A CF targets the soma of a Purkinje cell and ramifies into several tendril fibers that run along thick stem dendrites (low magnification views on left). The varicose swellings along the CF overlap with VGluT2-positive puncta indicating CF terminals (high magnification views on right). **B**: Confocal (left) and graphic (right) images of CFs and a multiplanar Purkinje cell at P18. Cell-1 and Cell-2 are associated by at least 2 and 3 CFs, respectively. The main ascending fibers are indicated by arrowheads in confocal images and by yellow in graphic images. The minor CFs apposing distal dendrites (asterisks in confocal images; light and dark pink in graphic images) innervate the soma of nearby Purkinje cells (circles in confocal images). Dendrites in minor planes are pseudocolored in blue and green in graphic images. Coronal views of graphic images on right indicate that both dendrites and CFs extend in multiple sagittal planes. **C**: A multiplanar Purkinje cell associated with multiple CFs. This Purkinje cell extends dendrites in three distinct sagittal planes (arrows in the coronal view) and receives inputs from at least three different CFs (asterisks, filled and blank arrowheads in the sagittal view). The boxed region in the left panel is enlarged in right panels. In addition to the ascending CF in the proximal part of the dendrite (asterisks), a CF from a different origin is closely apposed to the distal part of the dendrite (arrowheads). Triple staining suggests that both the main and minor CFs form VGluT2-positive synapses on the Purkinje dendrite. Scale bars: 20 µm in **B** and left panels in **A, C**; 10 µm in right panels in **A, C**.

BDA tracer was injected into the inferior olive of the AAV-injected mice at P14, and labeled axons innervating GFP-infected Purkinje cells were assayed at P18 or P25. Some CFs were found to associate with the soma or the proximal end of the primary stem dendrite of a GFP-labeled Purkinje cell (Figure 3A). Consistent with previous observations, the ascending CFs ramified into a few branches that twisted around thick Purkinje dendrites. The tendril fibers formed numerous varicosities that overlapped with punctate expression of VGluT2, a vesicular glutamate transporter specific to CF terminals in the molecular layer [36], [37]. Notably, the multiplanar Purkinje cells were often approached by multiple distinct CFs besides the major ascending fiber (Figure 3B). The minor CFs often appeared to appose to the soma of a nearby unlabeled Purkinje cell: they entered the molecular layer without contacting with the cell body of the labeled Purkinje cell and directly approached to its distal dendrites near the border of the CF innervation territory. In all cases examined, VGLuT2+ terminals made by minor CFs were on distal branchlets and isolated from those of the main CF on proximal shafts (Figure 3C). In a coronal view, the multiple CFs apposing to a Purkinje cell ran roughly in parallel along distinct sagittal planes. The dendrite tree in the main sagittal plane was mainly associated with the primary ascending CF, while those in minor sagittal planes were approached by both the primary CF and the terminals of other CFs (Figure S4A, B). The terminals of these minor CFs were apposed to the distal branches in minor planes and formed a few VGluT2-positive varicose swellings, suggesting that they may form presynaptic terminals on the distal dendrites (Figure 3C). Since not all CFs were labeled by dye injection, we could not examine whether multiplanar Purkinje cells were always associated with multiple CFs. However, even in the multiplanar cells that were apparently associated with a single tracer-labeled ascending CF, the arbors in minor sagittal planes were often apposed to VGluT2-positive puncta that were separate from the tracer-labeled ascending CF (Figure S4C). We also compared morphological CF association to multiplanar and monoplanar Purkinje cells. Almost all multiplanar Purkinje cells appeared to appose to multiple CFs, while a large majority of monoplanar Purkinje cells were associated with a single CF at P25 (Figure S5; multiple CF associations, 16 out of 19 multiplanar cells analyzed vs. 4 out of 16 monoplanar cells analyzed). The difference in apparent CF association between multiplanar and monoplanar cells at P25 as revealed by tracer labeling was statistically significant (p<0.001; Fisher's exact test). In contrast, Purkinje cells at P18 tend to be apposed to multiple CFs at distal dendrites regardless of their configurations (17 out of 22 multiplanar cells vs. 7 out of 12 monoplanar cells; p>0.1, Fisher's exact test).

Taken together, these results suggest a correlation of CF association and dendritic remodeling of the Purkinje cell.

### Purkinje cells in the sulcus retain multiplanar dendrites

**Figure 4:**
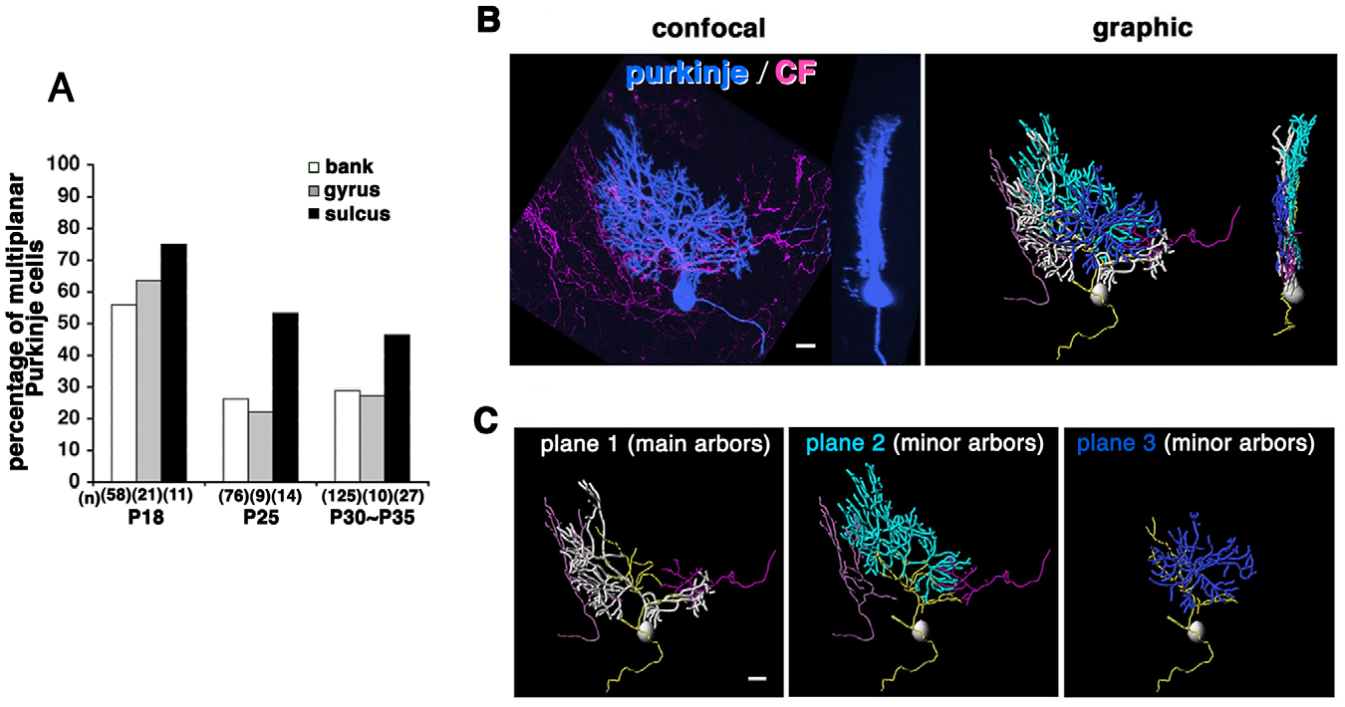
Purkinje cells are consistently multiplanar in the sulcus. **A**: The percentage of multiplanar Purkinje cells in distinct foliar subdivisions. The number of cells analyzed is indicated in parentheses. Remodeling from multiplanar to monoplanar dendrites is retarded in Purkinje cells in the sulcus. **B**: Confocal (left) and graphic (right) images of CFs and a multiplanar Purkinje cell in the sulcus. CFs in the sulcal region are radially arranged so that minor CFs tend to access the lateral side of the Purkinje cell. **C**: The Purkinje cell shown in **B** dissociated in three different sagittal planes. Scale bars: 20 µm.

In studying Purkinje cell morphology, we noticed that cells in the sulcal fundus often exhibited multiplanar arrangement even in adult stages. We thus compared dendritic configurations in three distinct subdivisions of the cerebellar folia (gyrus, bank and sulcus). Purkinje cells were arranged radially so that their dendrites densely overlapped with each other due to the concave shape of the molecular layer in the sulcus. Purkinje cells in the sulcus consistently exhibited multiplanar structures at higher percentage than in the gyrus and bank, and the difference was statistically significant (Figure 4A; P25–35; 48.8%, n = 41, sulcus vs. 23.9%, n = 201, bank; p<0.05; χ^2^ test). The multiplanar Purkinje cells in the sulcus were apposed to multiple CFs extended from the overlapping dendrites of other Purkinje cells (Figure 4B,C).

### Dendrite remodeling of Purkinje cells is impaired in mutant mice with aberrant afferent projections

**Figure 5:**
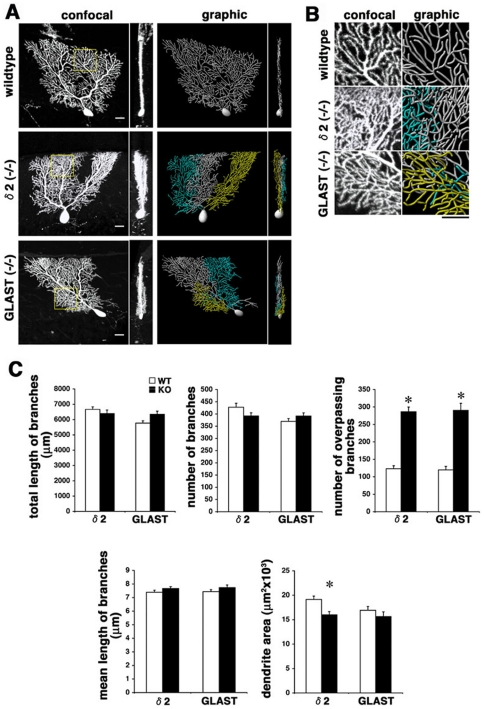
Impaired remodeling of Purkinje dendrites in GluRδ2- or GLAST-deficient mice. **A**: Cellular morphology of P30 Purkinje cells in wildtype, GluRδ2-deficient (δ2−/−) and GLAST-deficient (GLAST−/−) mice from sagittal (left panels) and coronal (right panels) views. The coronal views show irregular, multiplanar arrangement of dendrites in the GluRδ2−/− and GLAST−/− mice in contrast to the monoplanar appearance in the wildtype mouse. Dendrites in minor sagittal planes are pseudocolored in graphic images. **B**: High power sagittal views of dendrites in wildtype and mutant mice in respective boxed regions in **A**. Dendrites significantly overpass one another in sagittal views in GluRδ2−/− and GLAST−/− mice. **C**: Quantitative comparison of dendrite morphology of GluRδ2−/− and GLAST −/− Purkinje cells with respective wildtype littermates. Cells in the bank region of lobules IX and X were analyzed. n = 10 cells for each data point, mean±s.e.m., Student's t test, *p<0.01. Scale bars: 20 µm.

We next explored the conditions under which dendrite remodeling is impaired. We analyzed the dendritic configuration of Purkinje cells in GluRδ2-deficient mice to assess the correlation with normal Purkinje cell connectivity. Glutamate Receptor δ2 subunit is a member of the ionotropic glutamate receptor that is highly enriched in Purkinje cells. GluRδ2-deficient mice exhibit significant reduction in PF-Purkinje cell synapses, expansion of CF territory and ectopic CF innervation. Further, long-term depression of PF-Purkinje cell synapses, motor learning and motor coordination are impaired in GluRδ2-deficient mice [13], [38], [39]. In contrast to previous observation that Purkinje dendrite morphology was unaffected in GluRδ2-deficient mice, the monoplanar arborization was significantly disrupted in mutants as compared to wildtype littermates (Figure 5A, C; multiplanar cells; 22.3%, n = 112, P30 wildtype vs. 47.3%, n = 112, P30 GluRδ2-deficient mutant, p<0.01; χ^2^ test). From a sagittal view, dendritic branches significantly overpassed one another in GluRδ2-deficient mice (Figure 5B). Quantitative assessments revealed that GluRδ2 deficiency induced a marked increase in branch overpasses, and a slight decrease in the area occupied by the dendrite in a sagittal plane (Figure 5C). Other aspects of dendrite morphology including the number, total length of branches, and the mean branch length were not affected by GluRδ2 deficiency.

Dendritic configuration in Purkinje cells was also analyzed in GLAST null mutants. GLAST is a major glutamate transporter expressed in Bergmann glia that is responsible for removal of the released glutamate at CF-Purkinje cell synapses [40]. GLAST knockout mice exhibit motor discoordination caused by ectopic inputs to Purkinje dendrites from adjacent CF synapses of other Purkinje cells [24], [41]. Consistently, Purkinje cells in GLAST-deficient mice extended multiplanar dendrites in contrast to the monoplanar dendrites of the wildtype littermates at P30 (Figure 5A, B; multiplanar cells; 22.2%, n = 36, P30 wildtype vs. 48.7%, n = 115, P30 GLAST-deficient mutant; p<0.05; χ^2^ test). The branch overpasses were specifically increased despite no apparent abnormalities in overall dendrite development (Figure 5C).

Thus, monoplanar arborization of Purkinje cells was similarly disrupted in two lines of mutant mice with abnormal Purkinje cell connectivity.

### Abnormal activity of CFs disrupts dendritic remodeling of Purkinje cells

**Figure 6:**
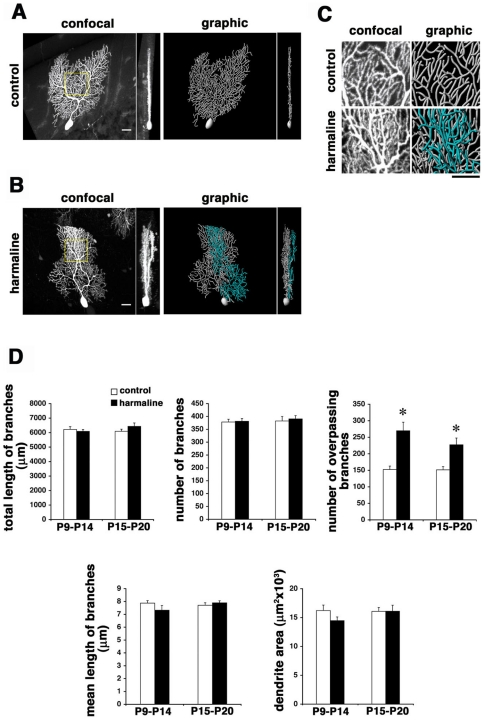
Disrupted remodeling of Purkinje dendrites in harmaline-treated mice. Cellular morphology of P30 Purkinje cells in mice treated with saline (**A**) and harmaline (**B**) at P9–P14. Dendrites become irregular and multiplanar by harmaline treatment compared to the control littermate. Dendrites in minor sagittal planes are pseudocolored in graphic images. **C**: High power sagittal views of dendrites in respective boxed regions in **A** and **B**. Dendrites significantly overpass one another in harmaline-treated mice. **D**: Quantitative comparison of dendrite morphology in mice treated with saline or harmaline during P9–P14 or P15–P20. Overpassing branches are significantly increased by harmaline treatment in either period. Cells in the bank region of lobules IV and V were analyzed. n = 10 for each data point, mean±s.e.m, Student's t test, *p<0.01. Scale bars: 20 µm.

We next examined the effect of abnormal afferent activity on Purkinje cell morphology. Systemic administration of harmaline induces a synchronous and enhanced firing of inferior olive neurons in mice [42], [43], [44]. It has also been shown that chronic application during the second postnatal week induces sustained multiple CF innervation in rats until adult ages [45]. We administered harmaline (30 mg/kg) by daily intraperitoneal injections for 6 days during the second (P9–P14) or the third (P15–P20) postnatal week, and assayed Purkinje cell morphology at P30. Harmaline treatment caused no gross abnormalities in mouse cerebellar cortex as previously reported [46]. We found that chronic application of harmaline during the second or the third postnatal week induced association of ectopic CFs in distal dendrites at P30 in mice (Figure S6). A majority of Purkinje cells in animals treated with harmaline either in the second or third postnatal week displayed multiplanar morphology at P30, while those in untreated control animals had mostly completed monoplanar rearrangement at this stage (Figure 6A, B; multiplanar cells; 27.1%, n = 96, control vs. 54.6%, n = 108, harmaline P9–P14, p<0.01; 25.3%, n = 75, control vs. 49.4%, n = 89, harmaline P15–P20, p<0.05; χ^2^ test). Overall dendritic growth was unaffected by harmaline treatment except for the striking increase in overpassing branches in a sagittal view (Figure 6C, D). These results strongly suggest that normal afferent inputs are critical for dendritic remodeling of Purkinje cells from multiplanar to monoplanar configurations.

## Discussion

We demonstrated that Purkinje cells undergo remodeling of dendrites from a multiplanar to a monoplanar configuration in late postnatal stages. Purkinje dendrites were often apposed with multiple CFs in the third postnatal week, and were then confined to single CF association concomitant with dendritic remodeling into a monoplanar arrangement. The remodeling of Purkinje dendrites was severely impaired in mutant mice where Purkinje dendrites receive aberrant afferent projections or by pharmacological disruption of normal afferent inputs. These results implicate afferent connectivity in patterning monoplanar dendrites of the Purkinje cell.

### Remodeling of Purkinje dendrites in late postnatal development

The murine cerebellar cortex undergoes dynamic developmental changes during the second and third postnatal weeks. These include the appearance and deepening of the fissures and lobules, gradual disappearance of the external granule layer, and enlargement of the molecular layer and the granule layer [35]. Since Purkinje dendrites show a rapid increase in size and complexity until P18, transient heterotopic extension of dendrites in a multiplanar arrangement might be a consequence of extensive arbor growth beyond the spatial limit of the growing molecular layer during the second and third postnatal week. Multiplanar dendrites might thus emerge more frequently in the sulcus than other subdivisions due to steric crowding of dendrites in the concave molecular layer in this region. Transient biplanar arrangement of Purkinje dendrites was also observed in the second postnatal week in cat cerebellum by previous Golgi impregnation studies [16]. Contrary to the previous suggestion that this morphological variation is attributed to species difference, the present results indicate that the remodeling of Purkinje cell from a multiplanar to a monoplanar arrangement during late postnatal development is fundamental in mammals.

How are the multiplanar dendrites rearranged in a single plane during the short interval between P18 and P25? One possibility is that dendrites in minor planes seen around P18 are selectively retracted and only those in the main sagittal planes are retained. Alternatively, the minor dendrites might be kept and translocated to fill spaces within the main sagittal plane. If the latter were the case, the sagittal-sectional area in the molecular layer covered by each Purkinje cell should significantly increase between P18 and P25. In fact, there was no increase in the area covered by dendrites but rather a significant decrease in the number of dendritic branches between P18 and P25 (Figure 2B, F). These static analyses suggest that dendritic stratification occurs via loss of arbors in minor planes. This will be more carefully illustrated by improved long-term imaging studies in the future.

### Dendritic remodeling and refinement of afferent connectivity

The second important finding is that dendritic remodeling of Purkinje cells is correlated with circuit assembly with afferent CF terminals. It is known that each Purkinje cell is innervated by multiple CFs in the first postnatal week, and activity-dependent elimination of minor CFs proceeds until monoinnervation is completed by P20–21 [47], [48]. We currently do not know if the ectopic CF associations to P18 dendrites evoke synaptic inputs, since previous electrophysiological studies have shown that more than 80% of Purkinje cells receive single CF input after P14. We surmise that the transient ectopic CF associations to multiplanar Purkinje cells at P18 are distinct from electrophysiologically identified multiple CF innervations in early postnatal life: Developmental elimination of multiple CF innervation has been shown to be initiated by activity-dependent competition of several equivalent CFs that share their innervation fields in the soma of a Purkinje cell in the first postnatal week [49], [50]; One of those CFs is selectively strengthened and extends from the soma toward dendrites, while other perisomatic CFs are mostly eliminated from the soma in the second postnatal week [51], [52]. This is in contrast to our observation that minor CFs are originated from ascending fibers of nearby Purkinje cells and directly accessed the distal dendrites of the multiplanar Purkinje cell in the third postnatal week (Figure 3). Thus, the transient ectopic apposition by adjacent CFs innervating nearby Purkinje cells in the third postnatal week is likely distinct from the perisomatic minor CFs seen in earlier stages. De novo addition of multiple CFs during the later phase is suggested by previous studies of GluRδ2-deficient mice in which supernumerary CFs arise from nearby Purkinje cells and contact small distal regions of the dendrite [39], [53]. It has also been shown that CFs retain structural and functional plasticity into adulthood [54], [55].

The flat arborization of Purkinje dendrites was severely impaired in GluRδ2 and GLAST mutants where Purkinje cell dendrites form aberrant circuits with multiple CFs. Abnormal activation of CFs by chronic application of harmaline during the second or third postnatal week also disrupted dendritic remodeling and ectopic CF elimination. Notably, dendritic remodeling was retarded in the sulcus of wildtype mice where Purkinje cells receive ectopic CF association (Figure 4)[29]. These results are in accordance with the hypothesis that maturation of the correct wiring of afferents is coupled with organization of Purkinje dendrites in monoplanar conformation. However, we currently do not know if CF association has instructive roles in dendrite remodeling. The mechanism underlying the local rearrangement of afferents and dendrites remains to be elucidated [56]. 

### The possible mechanisms and significance of dendritic configuration of Purkinje cells in the cerebellar circuit

CF axon terminals run along the flat Purkinje dendrites in a sagittal plane [12]. The monoplanar arborization would circumvent aberrant innervation from multiple adjacent CFs and thus provide a spatial framework for cerebellar neural circuits, which are coded in fine parasagittal subdivisions in the cerebellum. It would also serve to maximize the inputs from PFs. The activity-dependent refinement of dendrite configuration might contribute to optimization of local neural circuits during postnatal life.

On the other hand, the planarity of Purkinje dendrites is likely regulated by other mechanisms, since dendrites retain flatness along multiple sagittal planes in animals with abnormal CF innervation. Vertical contacts with PFs would be good candidate regulators of flat arborization of Purkinje dendrites as was suggested in earlier studies [33]. Likewise, interactions between branches of the same and adjacent Purkinje cells would also be involved in patterning the space-filling array of Purkinje dendrites with minimal overlaps and contacts (Figure 2A). The mechanisms of dendrite self-avoidance that ensure non-redundant coverage of the planar receptive field have been well studied in Drosophila sensory neurons and in murine retina [57], [58]. Analogous repulsive mechanisms might regulate non-overlapping and/or monoplanar branching of Purkinje dendrites. Analyses of dendritic configurations under various manipulations of afferent activities and molecules would address these hypotheses.

## Supporting Information

Figure S1 **Transduction of Purkinje cells in vivo with AAV-GFP.** **A**: Schematic drawing depicting virus injection site. AAV-GFP was injected in the molecular layer in the area of developing lobules IV–VI with a microsyringe. **B**: GFP fluorescence in a sagittal section of the cerebellar vermis at P30. AAV-GFP spread to all lobules and preferentially transduced Purkinje cells. Panels are oriented with the rostral side to the left. **C**: Magnified views of nearby labeled Purkinje cells at P26. A coronal view of 3D reconstruction shows parallel alignment of dendrites along the sagittal axis. Scale bars: 100 µm in **B**; 20 µm in **C**.
(TIF)

Figure S2 **Dendrite development in early postnatal weeks.** Confocal and graphic images of Purkinje cells at P7, P9 and P14. Respective sagittal (left) and coronal (right) views are shown. Remodeling from random, stellate dendrites to flat, oriented dendrites occurs between P7 and P9. Heterotopic dendrites extruded from the main sagittal plane of the stem dendrites are evident at P14 (pseudocolored in graphic images). Scale bars: 20 µm.
(TIF)

Figure S3 **Analyses of dendrite remodeling in tracer-labeled Purkinje cells.** Sagittal (left) and coronal (right) views of Purkinje cells at P18 (**A**) and P25 (**B**) labeled with BDA tracer. Arrows in (**B**) indicate background staining. The P18 Purkinje cell extends dendrites in multiple sagittal planes, while the P25 Purkinje cell arranges dendrites in a single sagittal plane, consistent with the results obtained by AAV-mediated expression of GFP. Scale bars, 20 µm. **C**: The proportion of multiplanar Purkinje cells as revealed by AAV-derived GFP and BDA labeling. The number of cells analyzed is indicated in parentheses. The results obtained by the two methods show no statistically significant difference (N.S. p>0.1; χ^2^ test). The percentages of multiplanar cells are significantly different between P18 and P25 (** p<0.001; χ^2^ test).
(TIF)

Figure S4 Multiple CF innervation to dendritic arbors in distinct sagittal planes. **A**: AAV-GFP-infected Purkinje cell (pseudocolored in blue) receiving inputs from multiple CFs (magenta) shown in Fig. 3C. This Purkinje cell extends dendrites in three distinct sagittal planes (white, light and dark blue in graphic images on the right) and receives inputs from at least three different CFs (asterisks, filled and blank arrowheads in the confocal image on the left; also indicated by yellow, light and dark pink in graphic images). **B**: The Purkinje cell shown in **A** dissociated in three different sagittal planes. The main dendritic arbors in plane 1 (white) are only associated with the ascending CF (yellow). The minor dendritic arbors in plane 2 (light blue) and plane 3 (dark blue) are associated with respective minor CFs (light and dark pink) in addition to the ascending CF (yellow). **C**: A multiplanar Purkinje cell at P18 associated with only one BDA-labeled CF. The boxed region in the upper panel is enlarged in lower panels (double-, triple-staining and graphic images). Asterisks indicate the serial vesicular glutamate transporter VGluT2-positive puncta that are closely apposed to the tips of dendrites independent of the labeled ascending CF (arrowheads in confocal and graphic images; also indicated by yellow in graphic image). Scale bars: 20 µm.
(TIF)

Figure S5 **Mono-CF innervation in monoplanar Purkinje cells.** **A**: Confocal images of a monoplanar Purkinje cell (green), CFs (blue), and VGluT2 (magenta) at P25. The main ascending fiber is indicated by arrowheads. Scale bar, 20 µm. **B**: Graphic images of the CFs and Purkinje cell shown in **A**. The main ascending CF (yellow) innervates the monoplanar dendrites (white). **C**: A coronal view of the Purkinje cell and juxtaposing CFs. Except for the main ascending CF (yellow), none of other adjacent CFs (pseudocolored in red, green and pink) contact the Purkinje dendrites (white). **D**: The proportion of Purkinje cells innervated by multiple CFs. BDA-labeled CFs bearing vGluT2-positive terminals associating with GFP-labeled Purkinje dendrites were counted. The majority of Purkinje cells are associated by multiple CFs regardless of dendrite configuration at P18. In contrast, a large majority of monoplanar Purkinje cells receive inputs from a single CF, while almost all multiplanar cells receive multiple CF inputs at P25.
(TIF)

Figure S6 **Persistent multiple CF innervation induced by chronic application of harmaline.** **A**: Confocal and graphic images of an AAV-GFP-infected Purkinje cell and CFs in P30 mice treated with harmaline between P9–P14. Triple fluorescence for Purkinje cells (pseudocolored in blue), BDA-labeled CFs (magenta) and vesicular glutamate transporter VGluT2 (green) is shown. This Purkinje cell is apposed with at least two different CFs (yellow and pink in graphic images). **B**: High power views of dendritic arbors in the boxed region in **A**. In addition to the ascending CF in the proximal part of the dendrite (arrowheads), a CF of different origin is closely apposed to the distal part of the dendrite (asterisks). Both the main and minor CFs form VGluT2-positive synapses on the Purkinje dendrite. **C**: The Purkinje cell shown in **A** dissociated in two different sagittal planes. The main dendritic arbors in plane 1 (white) are only associated with the ascending CF (yellow). The minor dendritic arbors in plane 2 (blue) are associated with both the ascending and minor CFs (yellow and pink, respectively). Scale bars: 20 µm in **A, C**; 10 µm in **B**.
(TIF)

Video S1 **Rotational 3D movie of P6 Purkinje cell.**
(AVI)

Video S2 **Rotational 3D movie of P9 Purkinje cell.**
(AVI)

Video S3 **Rotational 3D movie of P18 Purkinje cell.**
(AVI)

Video S4 **Rotational 3D movie of P22 Purkinje cell.**
(AVI)
