# Supplementary figures and images for: Remodeling of Monoplanar Purkinje Cell Dendrites during Cerebellar Circuit Formation
Source: PLoS One. 2011 May 31;6(5):e20108. doi: 10.1371/journal.pone.0020108 (PMC3105010; doi:10.1371/journal.pone.0020108)

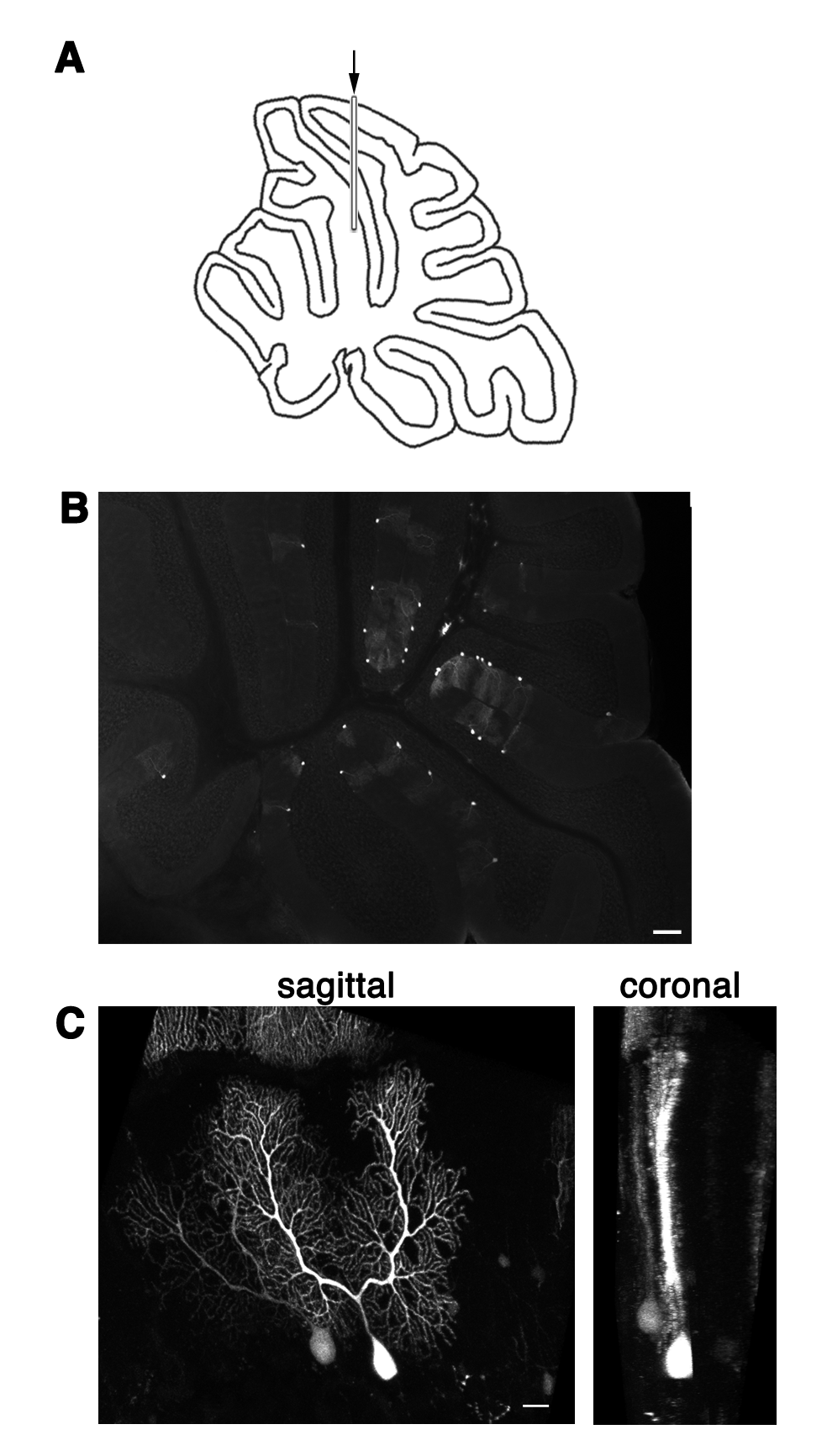

Supplement: Figure S1 — Transduction of Purkinje cells in vivo with AAV-GFP. A: Schematic drawing depicting virus injection site. AAV-GFP was injected in the molecular layer in the area of developing lobules IV–VI with a microsyringe. B: GFP fluorescence in a sagittal section of the cerebellar vermis at P30. AAV-GFP spread to all lobules and preferentially transduced Purkinje cells. Panels are oriented with the rostral side to the left. C: Magnified views of nearby labeled Purkinje cells at P26. A coronal view of 3D reconstruction shows parallel alignment of dendrites along the sagittal axis. Scale bars: 100 µm in B; 20 µm in C. (TIF) [file pone.0020108.s001.tif]

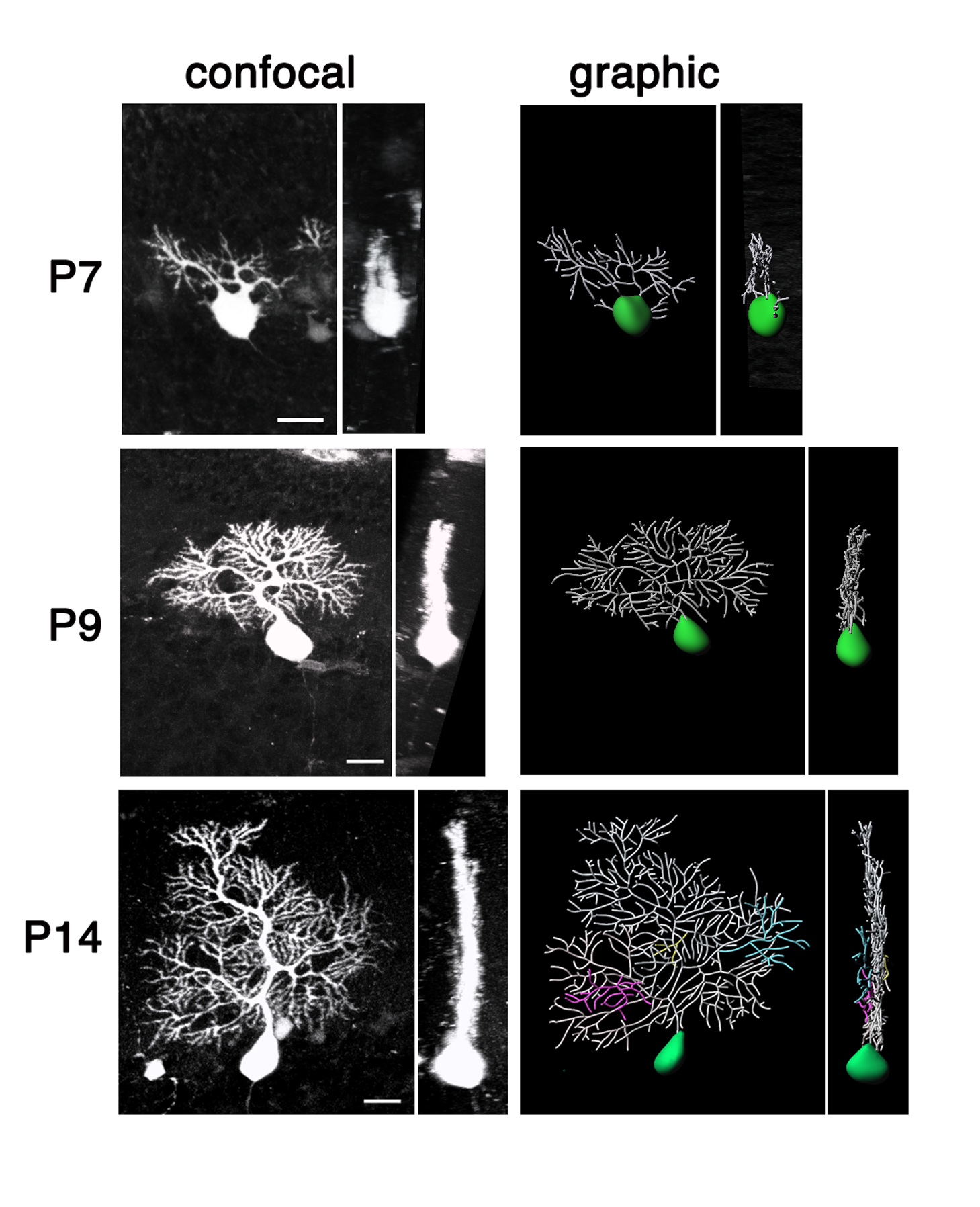

Supplement: Figure S2 — Dendrite development in early postnatal weeks. Confocal and graphic images of Purkinje cells at P7, P9 and P14. Respective sagittal (left) and coronal (right) views are shown. Remodeling from random, stellate dendrites to flat, oriented dendrites occurs between P7 and P9. Heterotopic dendrites extruded from the main sagittal plane of the stem dendrites are evident at P14 (pseudocolored in graphic images). Scale bars: 20 µm. (TIF) [file pone.0020108.s002.tif]

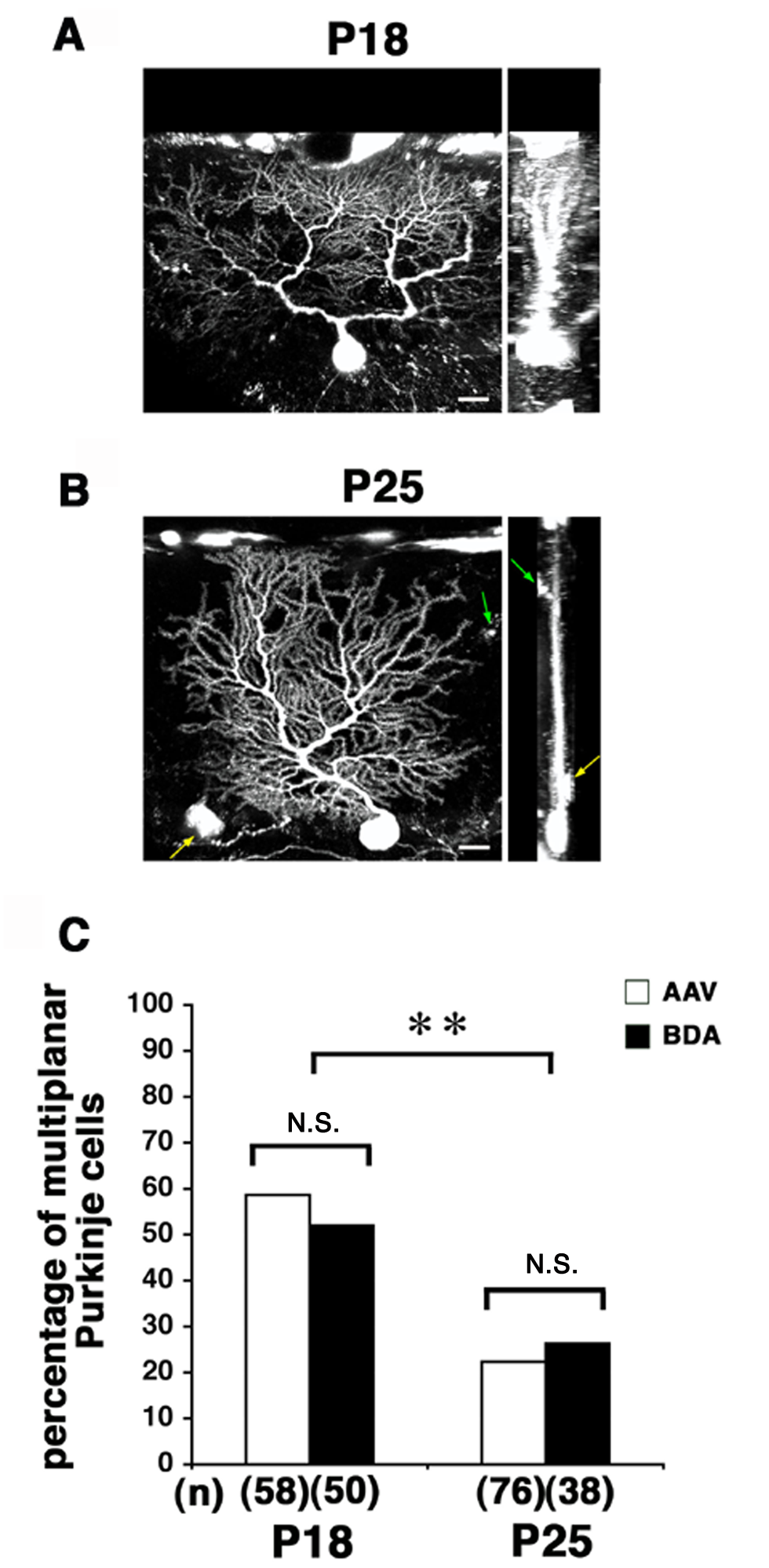

Supplement: Figure S3 — Analyses of dendrite remodeling in tracer-labeled Purkinje cells. Sagittal (left) and coronal (right) views of Purkinje cells at P18 (A) and P25 (B) labeled with BDA tracer. Arrows in (B) indicate background staining. The P18 Purkinje cell extends dendrites in multiple sagittal planes, while the P25 Purkinje cell arranges dendrites in a single sagittal plane, consistent with the results obtained by AAV-mediated expression of GFP. Scale bars, 20 µm. C: The proportion of multiplanar Purkinje cells as revealed by AAV-derived GFP and BDA labeling. The number of cells analyzed is indicated in parentheses. The results obtained by the two methods show no statistically significant difference (N.S. p>0.1; χ2 test). The percentages of multiplanar cells are significantly different between P18 and P25 (** p<0.001; χ2 test). (TIF) [file pone.0020108.s003.tif]

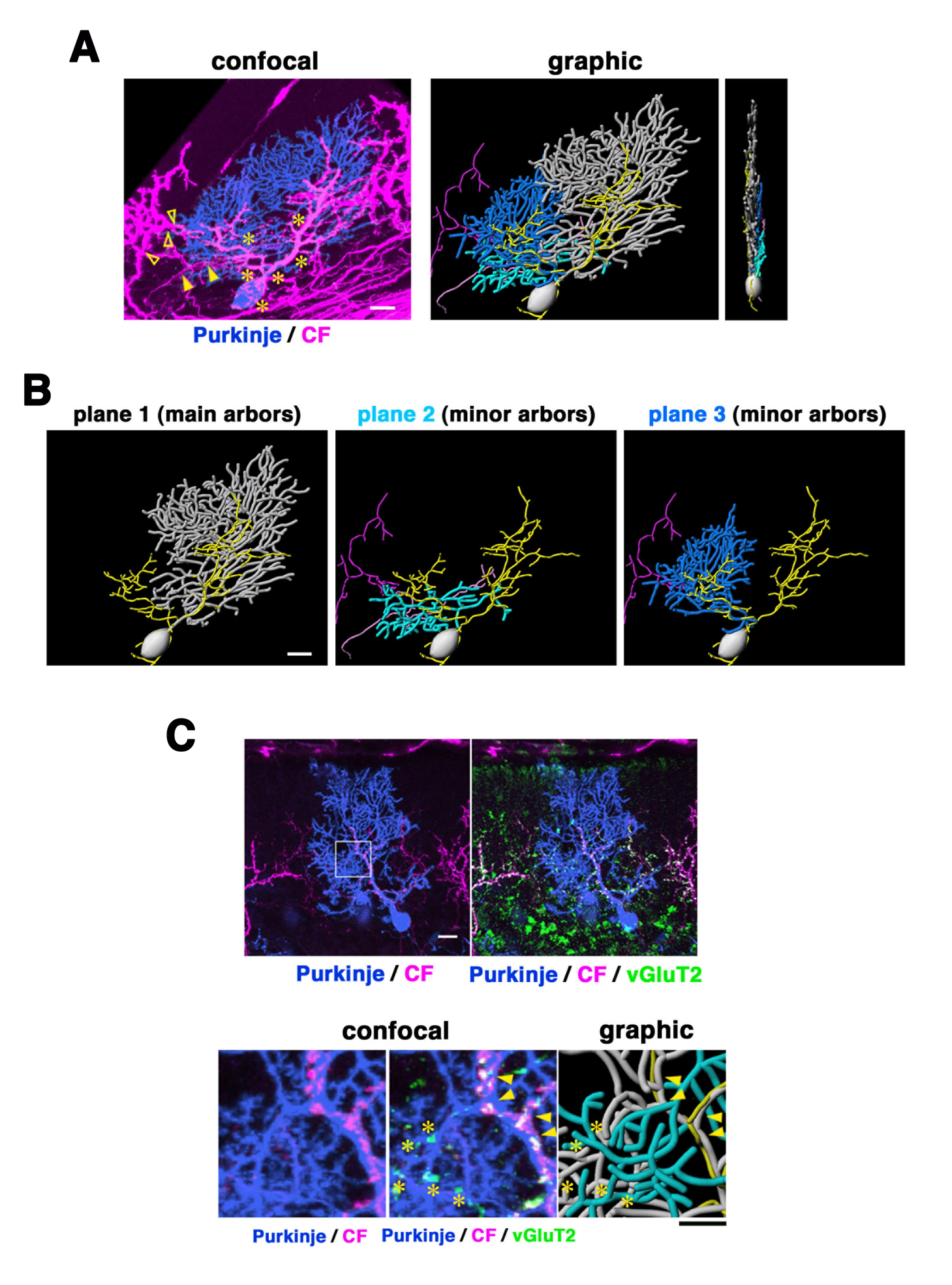

Supplement: Figure S4 — Multiple CF innervation to dendritic arbors in distinct sagittal planes. A: AAV-GFP-infected Purkinje cell (pseudocolored in blue) receiving inputs from multiple CFs (magenta) shown in Fig. 3C. This Purkinje cell extends dendrites in three distinct sagittal planes (white, light and dark blue in graphic images on the right) and receives inputs from at least three different CFs (asterisks, filled and blank arrowheads in the confocal image on the left; also indicated by yellow, light and dark pink in graphic images). B: The Purkinje cell shown in A dissociated in three different sagittal planes. The main dendritic arbors in plane 1 (white) are only associated with the ascending CF (yellow). The minor dendritic arbors in plane 2 (light blue) and plane 3 (dark blue) are associated with respective minor CFs (light and dark pink) in addition to the ascending CF (yellow). C: A multiplanar Purkinje cell at P18 associated with only one BDA-labeled CF. The boxed region in the upper panel is enlarged in lower panels (double-, triple-staining and graphic images). Asterisks indicate the serial vesicular glutamate transporter VGluT2-positive puncta that are closely apposed to the tips of dendrites independent of the labeled ascending CF (arrowheads in confocal and graphic images; also indicated by yellow in graphic image). Scale bars: 20 µm. (TIF) [file pone.0020108.s004.tif]

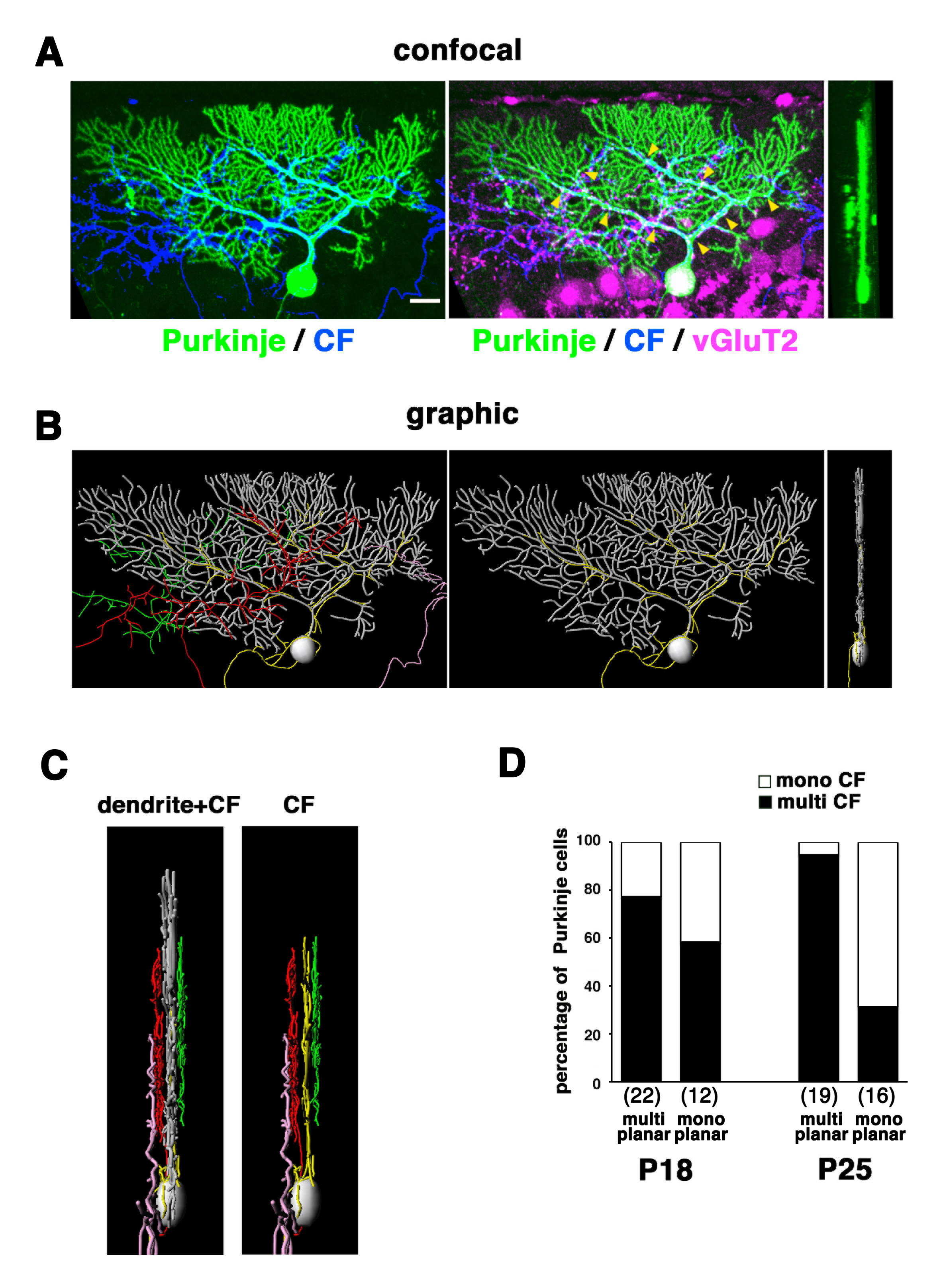

Supplement: Figure S5 — Mono-CF innervation in monoplanar Purkinje cells. A: Confocal images of a monoplanar Purkinje cell (green), CFs (blue), and VGluT2 (magenta) at P25. The main ascending fiber is indicated by arrowheads. Scale bar, 20 µm. B: Graphic images of the CFs and Purkinje cell shown in A. The main ascending CF (yellow) innervates the monoplanar dendrites (white). C: A coronal view of the Purkinje cell and juxtaposing CFs. Except for the main ascending CF (yellow), none of other adjacent CFs (pseudocolored in red, green and pink) contact the Purkinje dendrites (white). D: The proportion of Purkinje cells innervated by multiple CFs. BDA-labeled CFs bearing vGluT2-positive terminals associating with GFP-labeled Purkinje dendrites were counted. The majority of Purkinje cells are associated by multiple CFs regardless of dendrite configuration at P18. In contrast, a large majority of monoplanar Purkinje cells receive inputs from a single CF, while almost all multiplanar cells receive multiple CF inputs at P25. (TIF) [file pone.0020108.s005.tif]

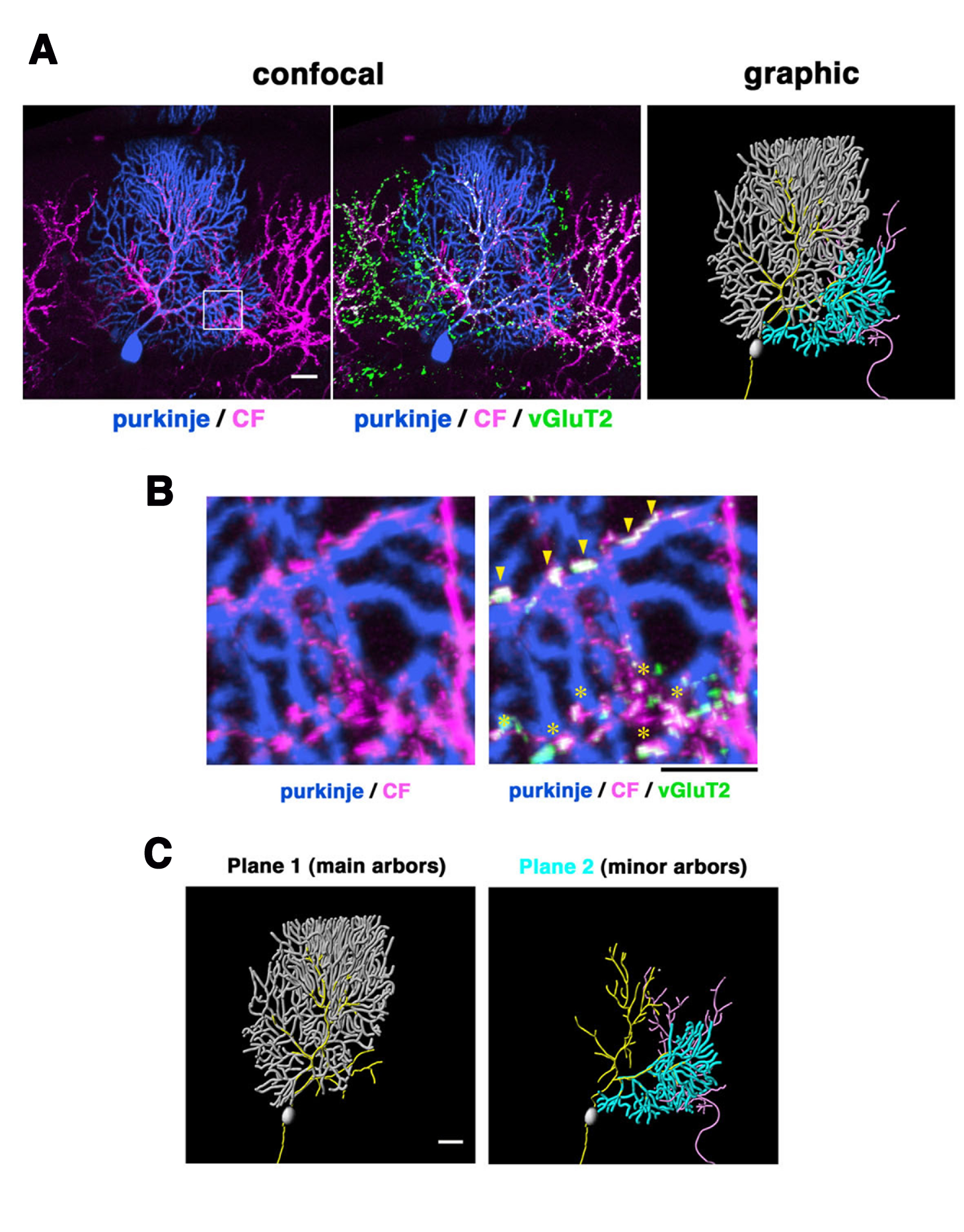

Supplement: Figure S6 — Persistent multiple CF innervation induced by chronic application of harmaline. A: Confocal and graphic images of an AAV-GFP-infected Purkinje cell and CFs in P30 mice treated with harmaline between P9–P14. Triple fluorescence for Purkinje cells (pseudocolored in blue), BDA-labeled CFs (magenta) and vesicular glutamate transporter VGluT2 (green) is shown. This Purkinje cell is apposed with at least two different CFs (yellow and pink in graphic images). B: High power views of dendritic arbors in the boxed region in A. In addition to the ascending CF in the proximal part of the dendrite (arrowheads), a CF of different origin is closely apposed to the distal part of the dendrite (asterisks). Both the main and minor CFs form VGluT2-positive synapses on the Purkinje dendrite. C: The Purkinje cell shown in A dissociated in two different sagittal planes. The main dendritic arbors in plane 1 (white) are only associated with the ascending CF (yellow). The minor dendritic arbors in plane 2 (blue) are associated with both the ascending and minor CFs (yellow and pink, respectively). Scale bars: 20 µm in A, C; 10 µm in B. (TIF) [file pone.0020108.s006.tif]
